# Supplementary material for: In vitro anti-diabetic effects and phytochemical profiling of novel varieties of Cinnamomum zeylanicum (L.) extracts
Source: PeerJ. 2020 Nov 2;8:e10070. doi: 10.7717/peerj.10070 (PMC7643550; doi:10.7717/peerj.10070)
Supplement: Supplemental Information 1 [file peerj-08-10070-s001.docx]

***In vitro* anti-diabetic effects and phytochemical profiling of novel varieties of**

***Cinnamomum zeylanicum* (L.) extracts**

**GCMS profile of various types of cinnamon extracts**


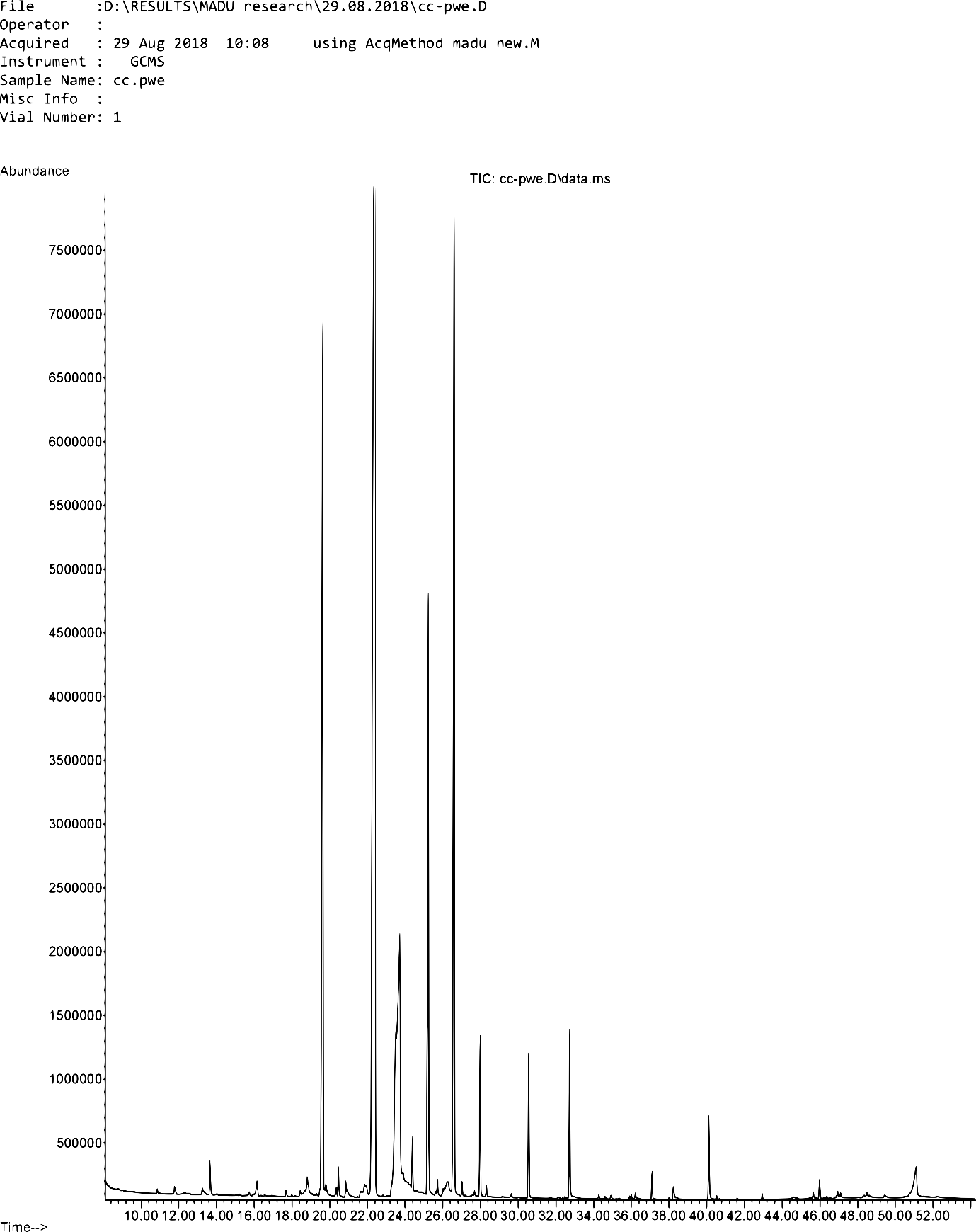


***Figure 1:*** *GCMS profile of commercially available C. zeylanicum by pressurize water extracts*


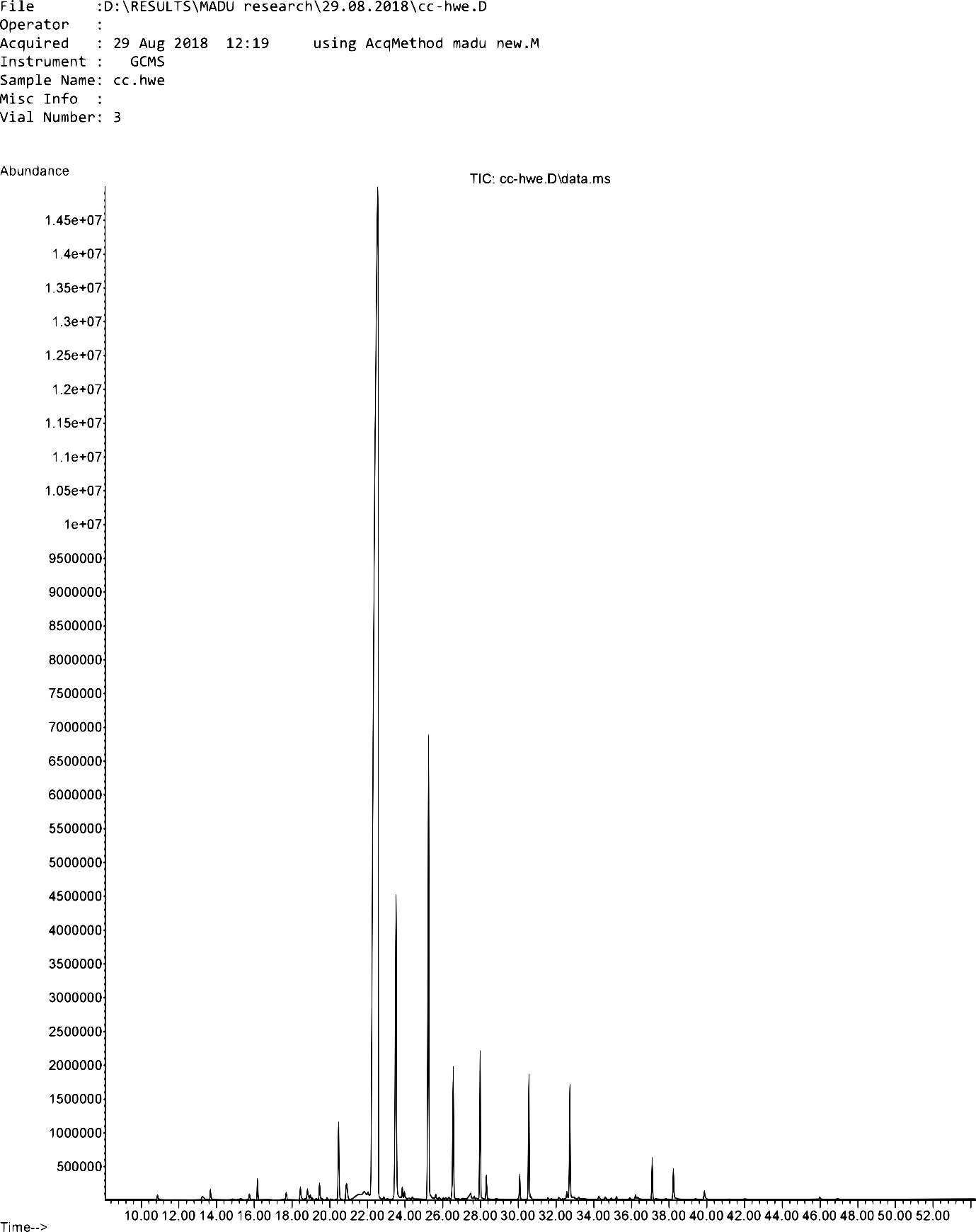


***Figure 2:*** *GCMS profile of commercially available C. zeylanicum by hot water extracts*


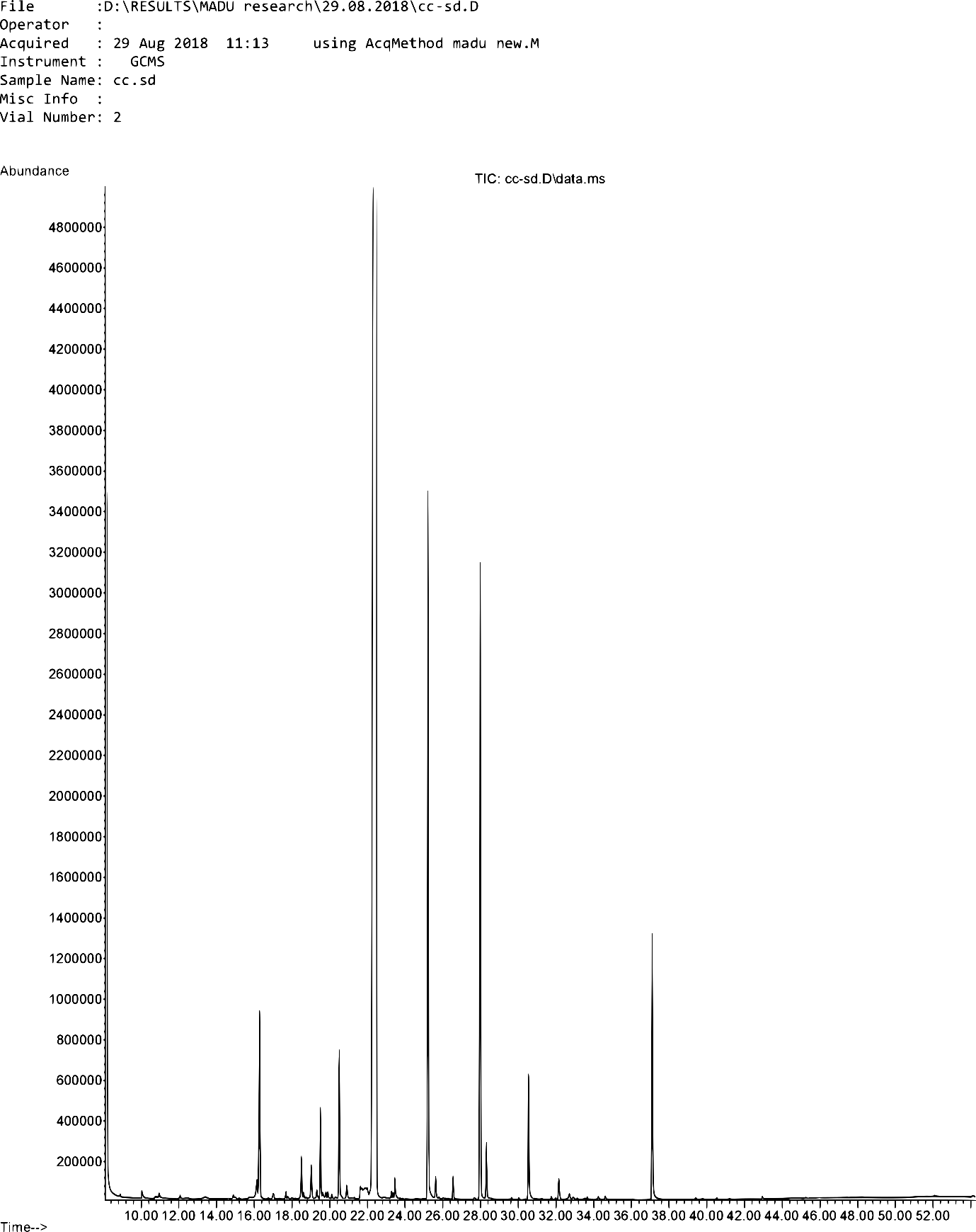


***Figure 3:*** *GCMS profile of commercially available C. zeylanicum by steam distillation*


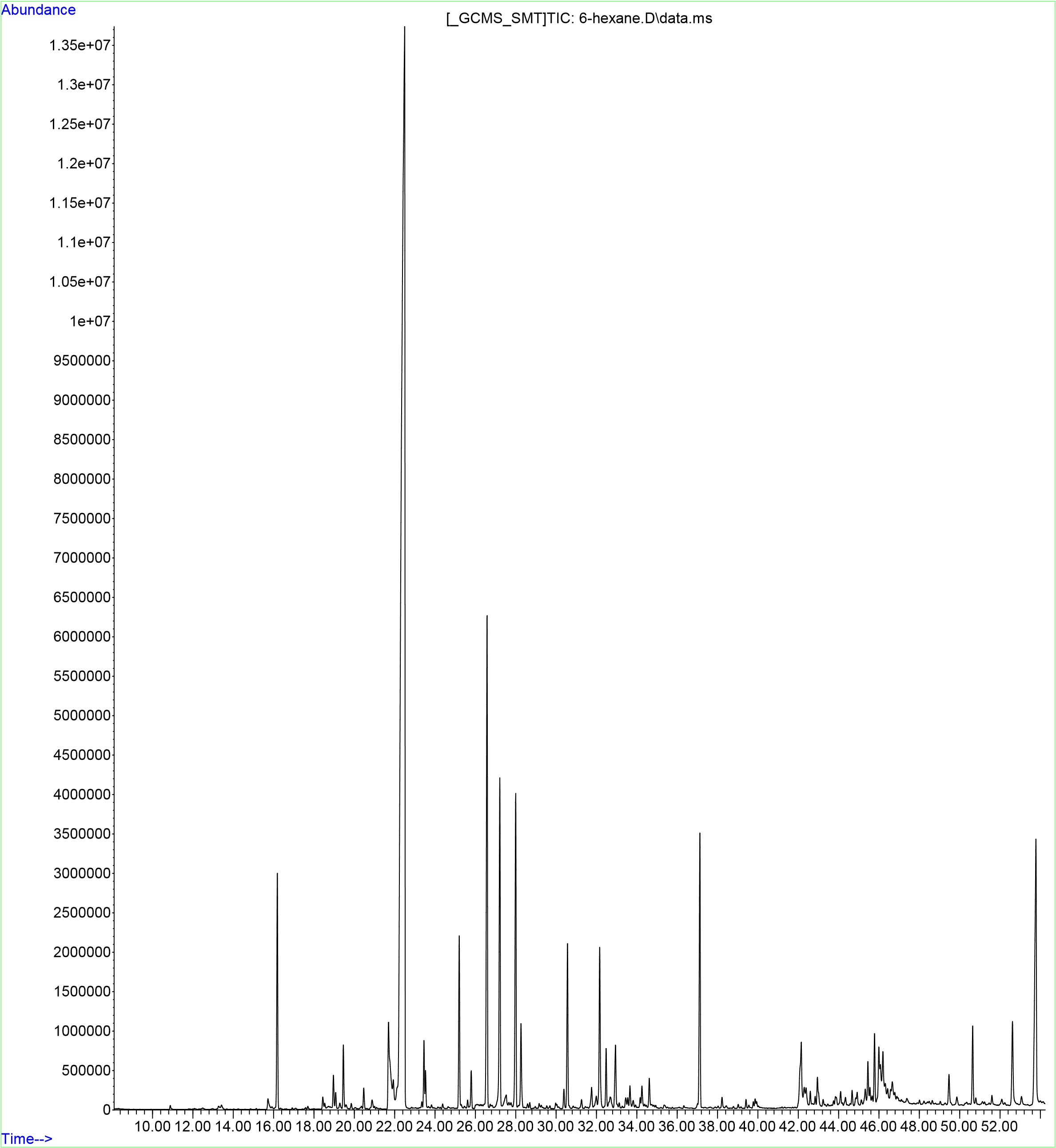


***Figure 4:*** *GCMS profile of commercially available C. zeylanicum variety solvent extraction*


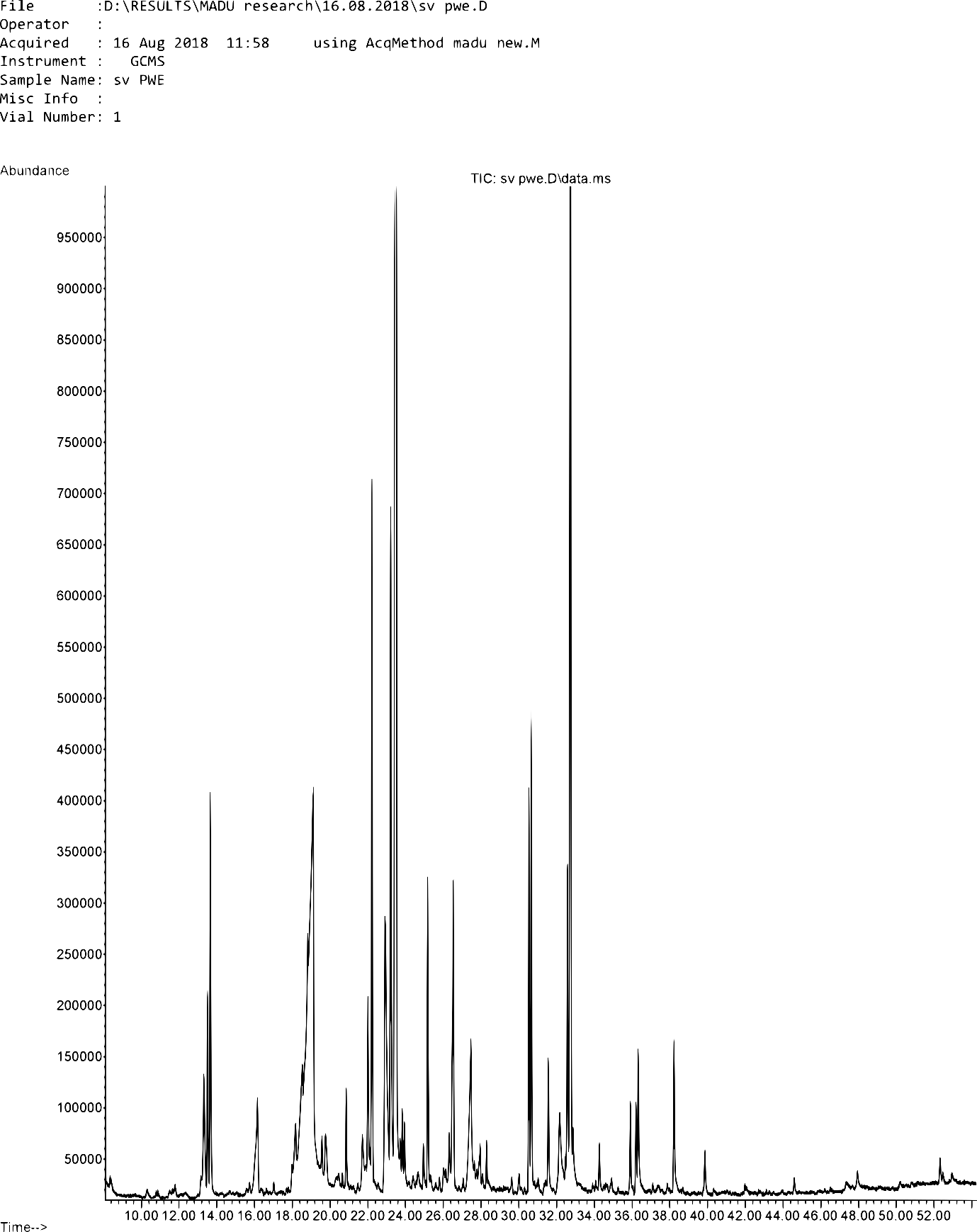


***Figure 5:*** *GCMS profile of C. zeylanicum (Sri Wijaya variety) by pressurized water extracts*


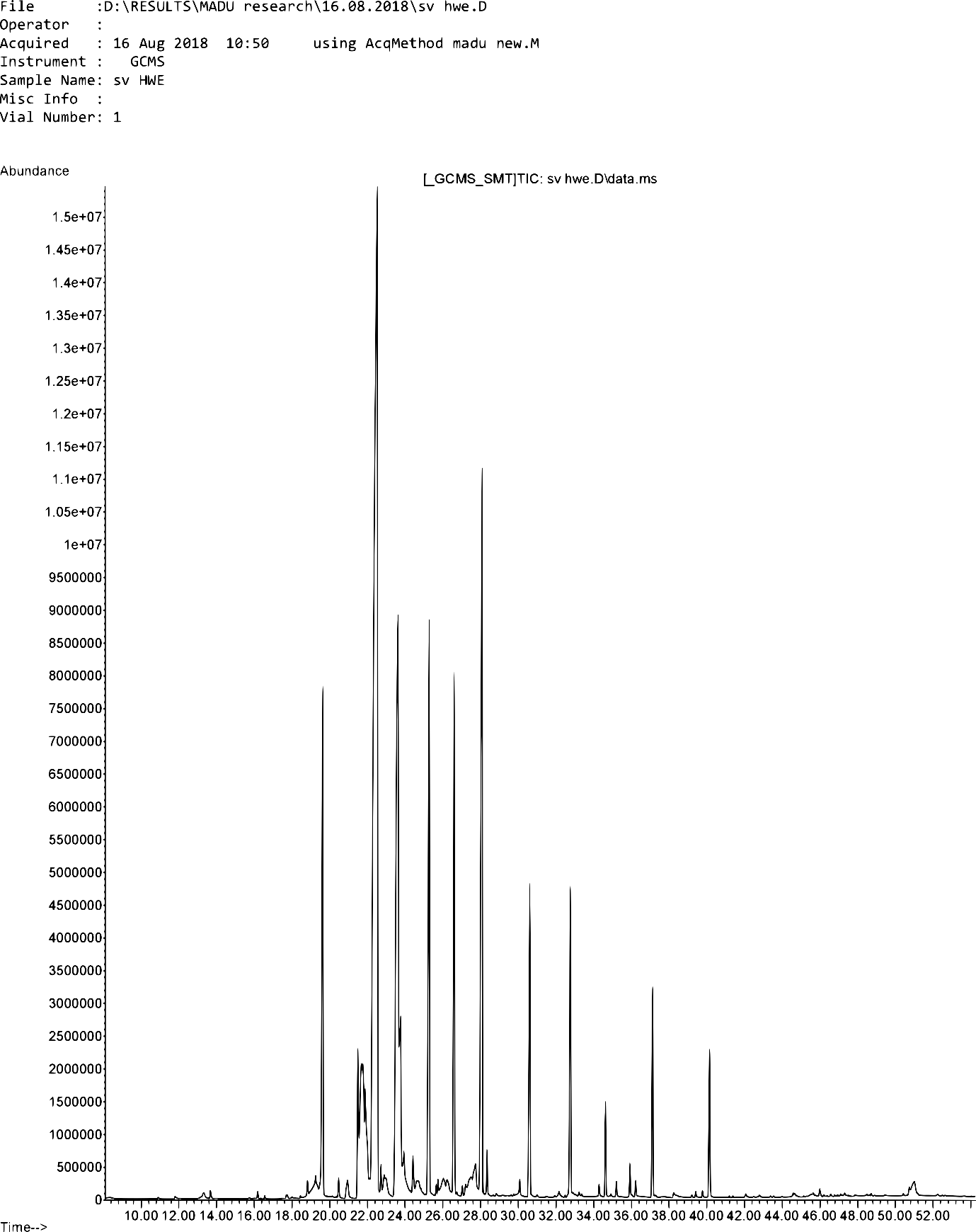


***Figure 6:*** *GCMS profile of C. zeylanicum (Sri Wijaya variety) by hot water extracts*


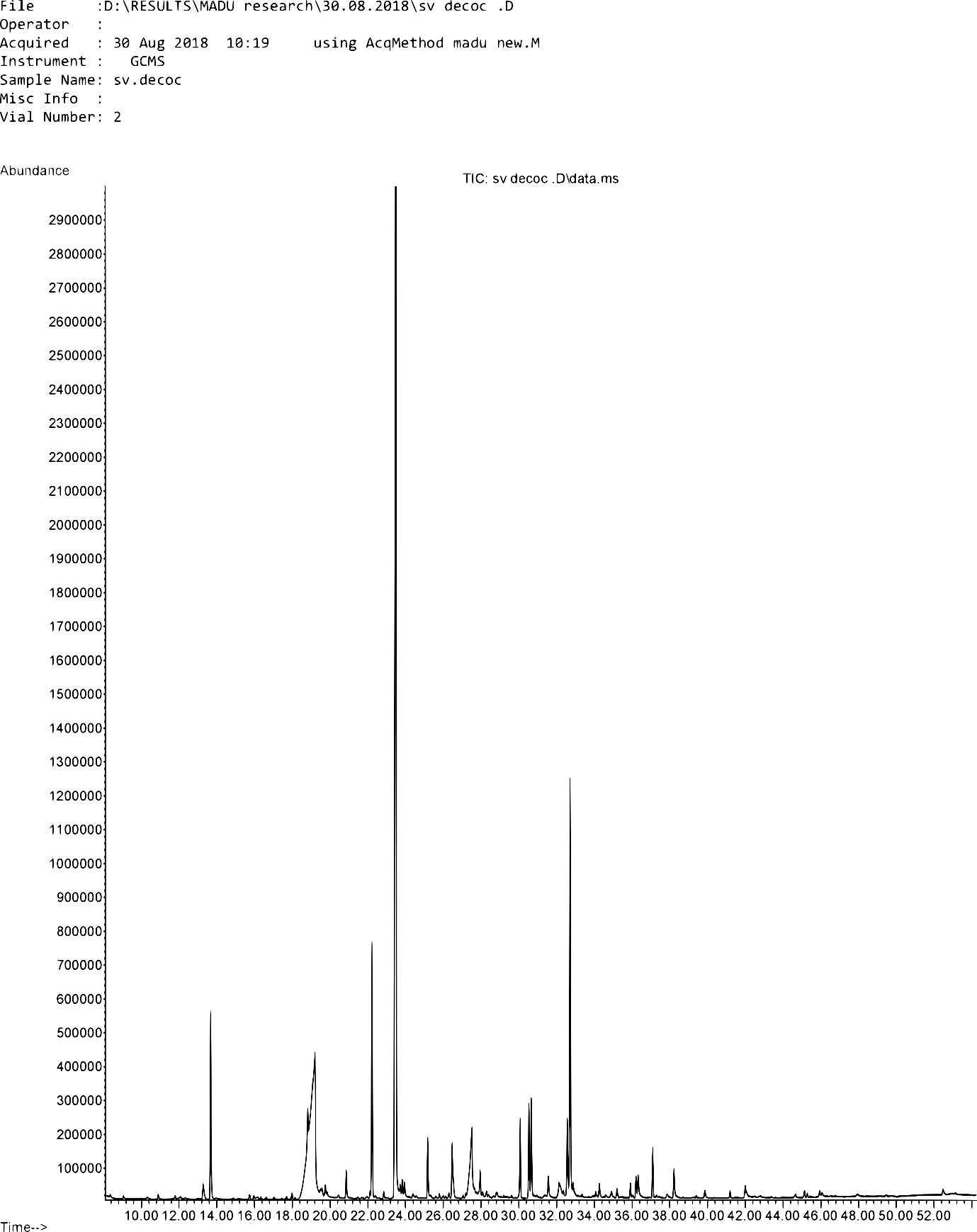


***Figure 7:*** *GCMS profile of C. zeylanicum (Sri Wijaya variety) by decoction water extracts*


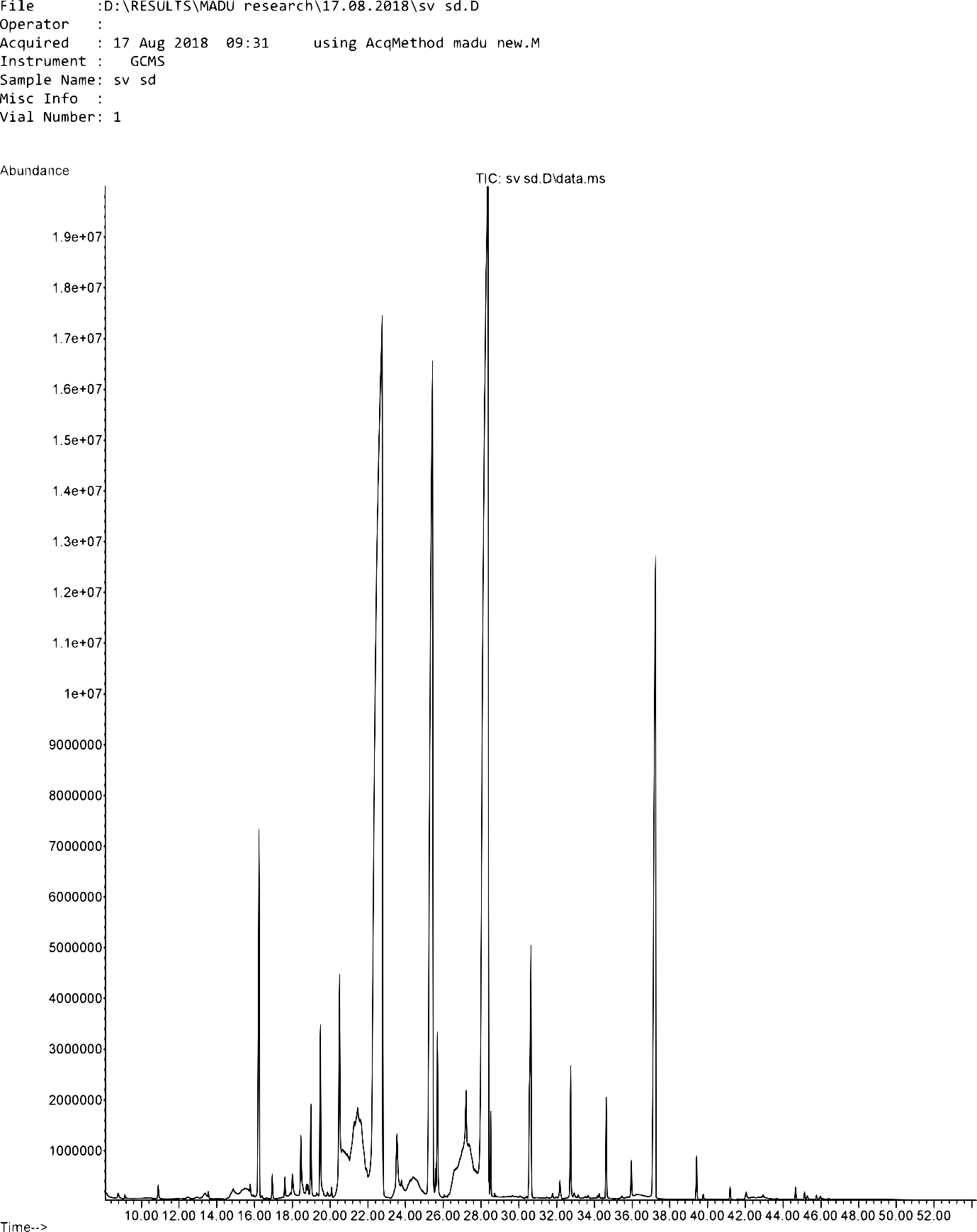


***Figure 8:*** *GCMS profile of C. zeylanicum (Sri Wijaya variety) by steam distillation*


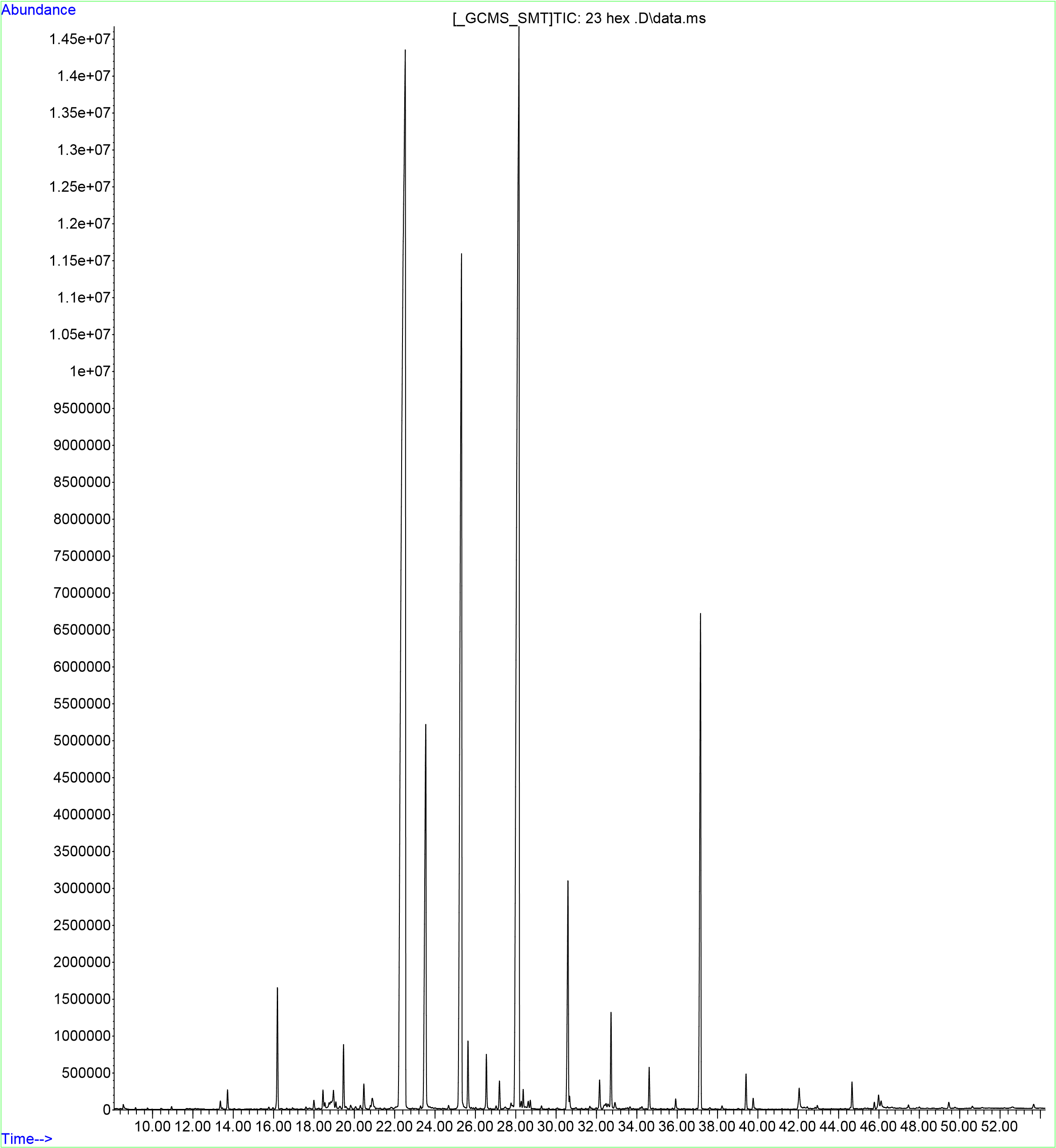


***Figure 9:*** *GCMS profile of C. zeylanicum (Sri Wijaya variety) solvent extraction*


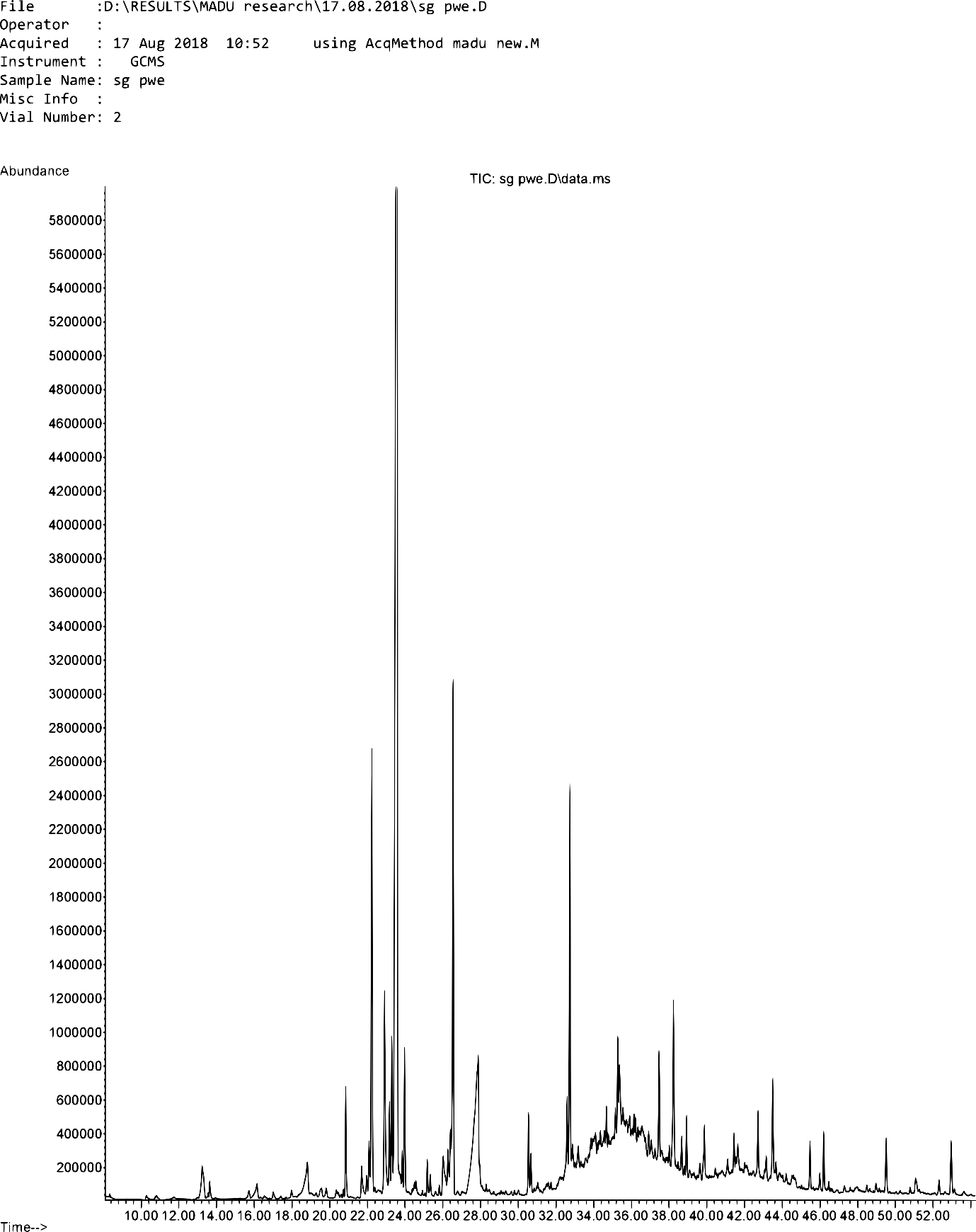


***Figure 10:*** *GCMS profile of C. zeylanicum (Sri Gemunu variety) by pressurized water extracts*


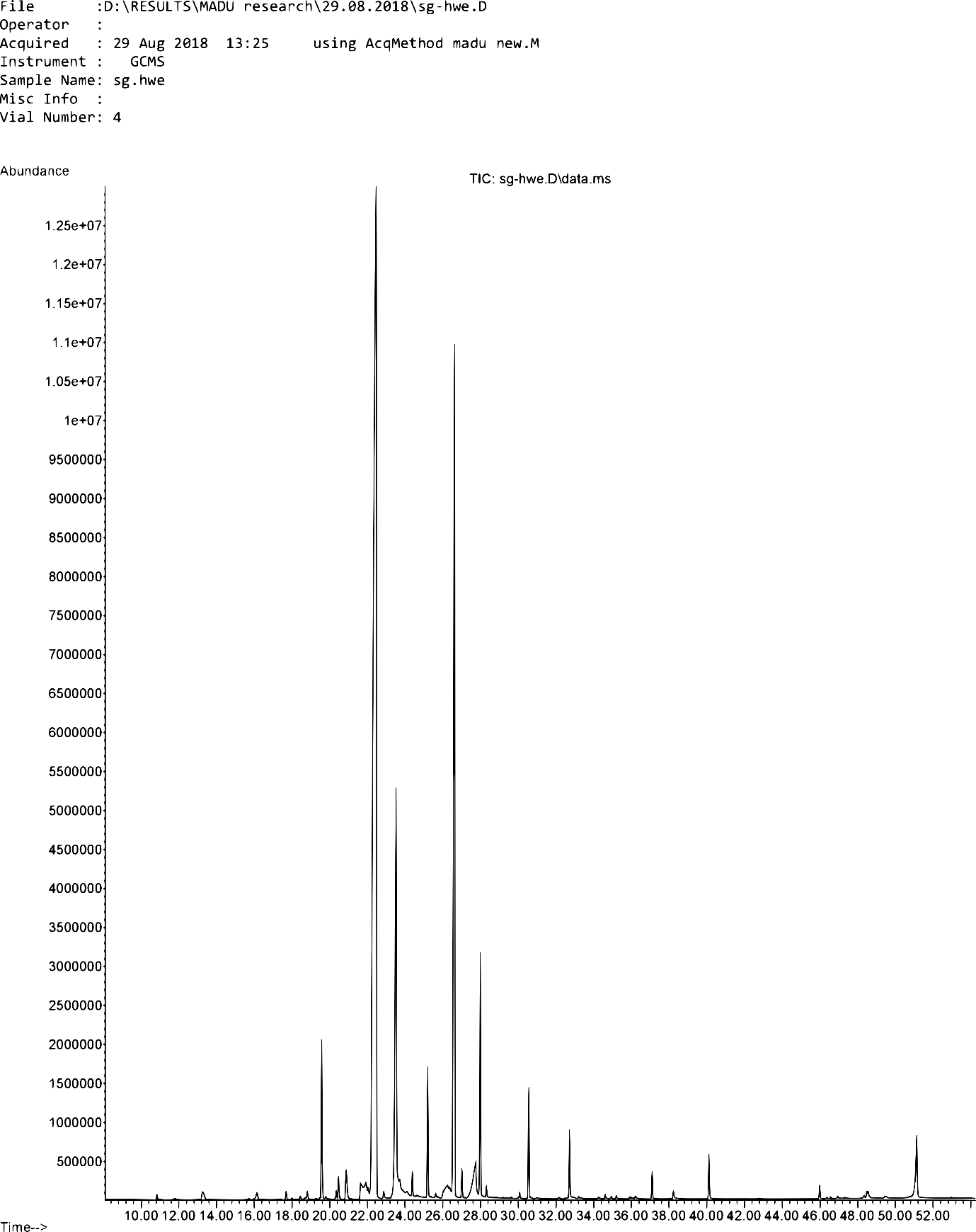


***Figure 11:*** *GCMS profile of C. zeylanicum (Sri Gemunu variety) by hot water extracts*


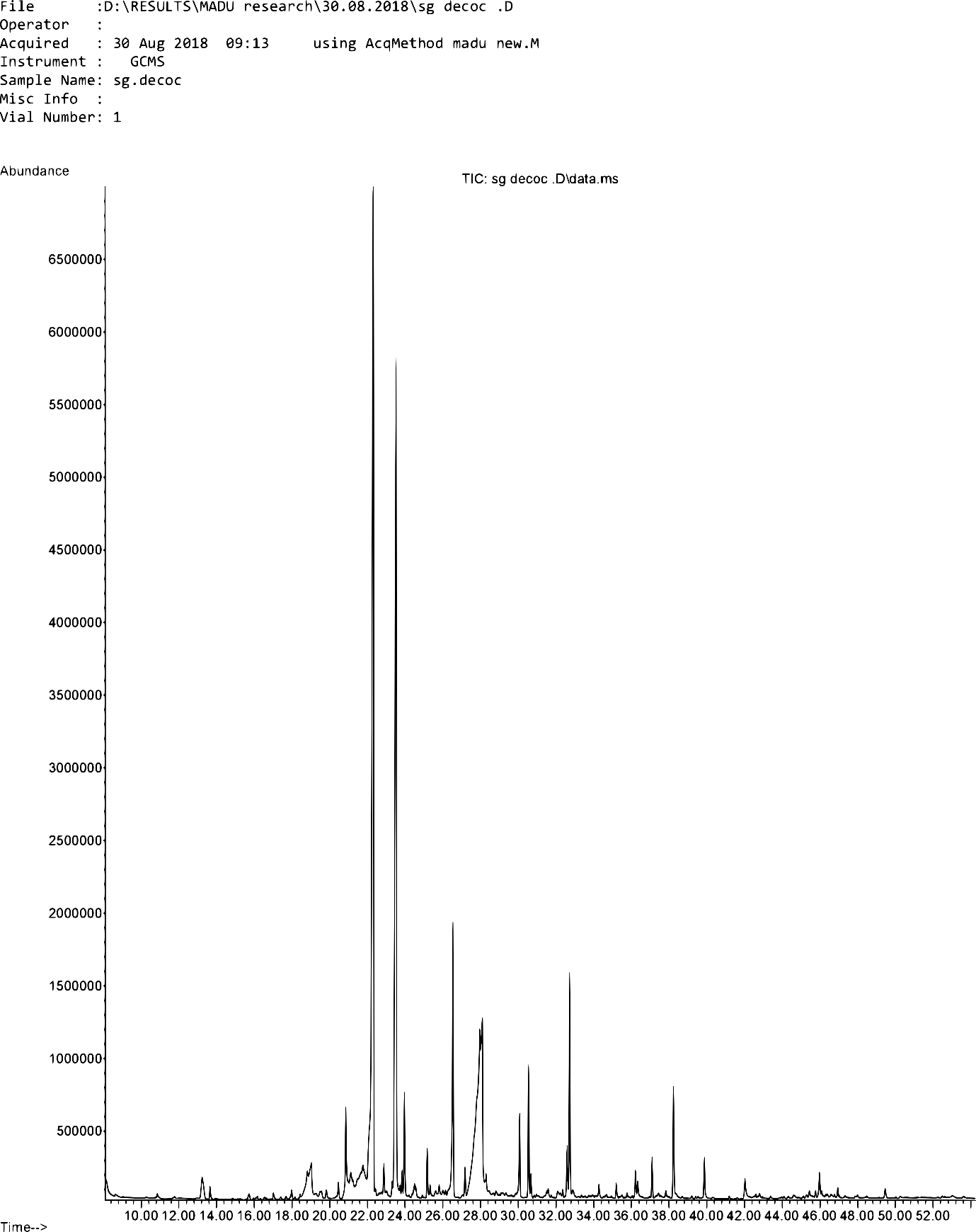


***Figure 12:*** *GCMS profile of C. zeylanicum (Sri Gemunu variety) by decoction water extracts*


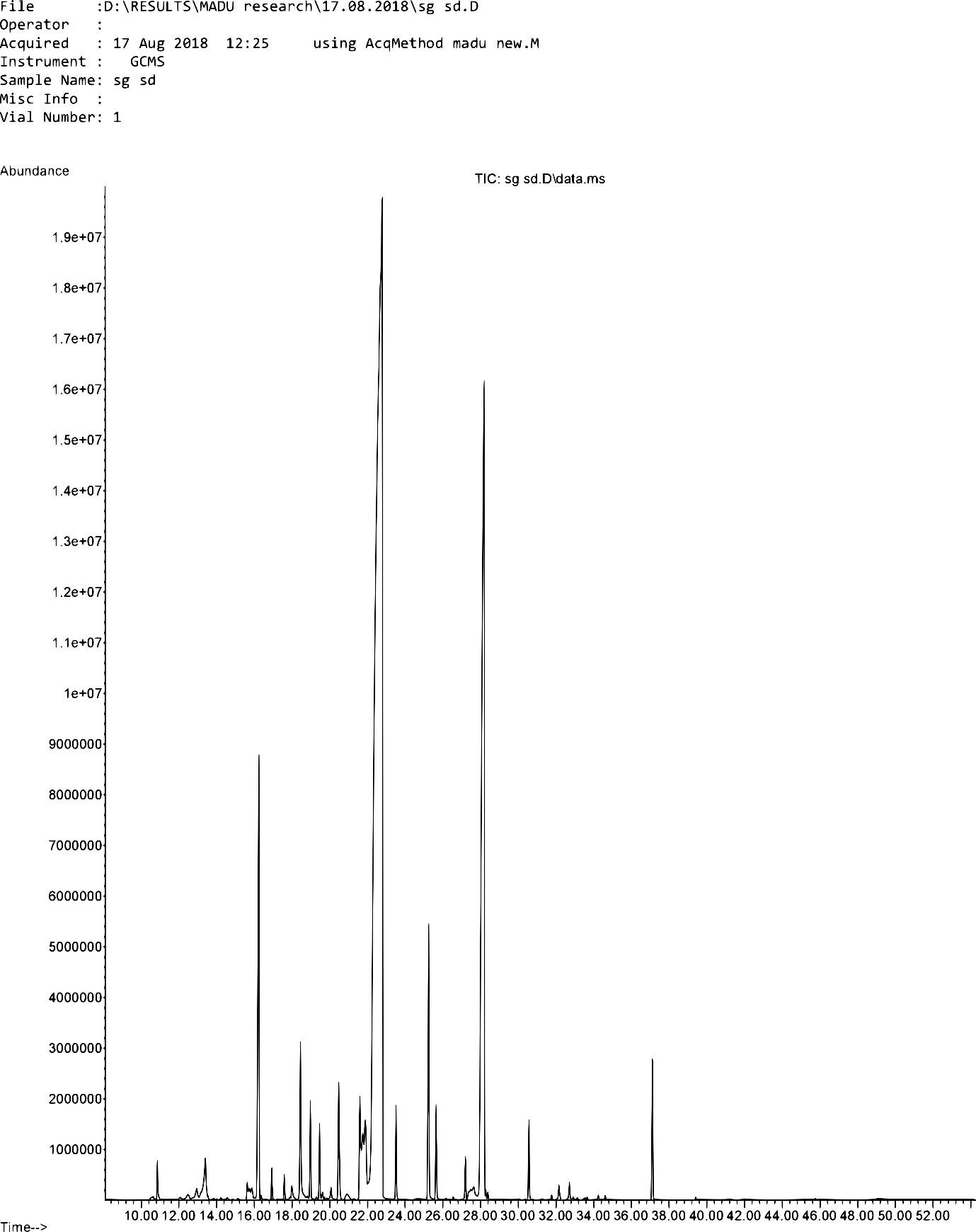


***Figure 13:*** *GCMS profile of C. zeylanicum (Sri Gemunu variety) by steam distillation*


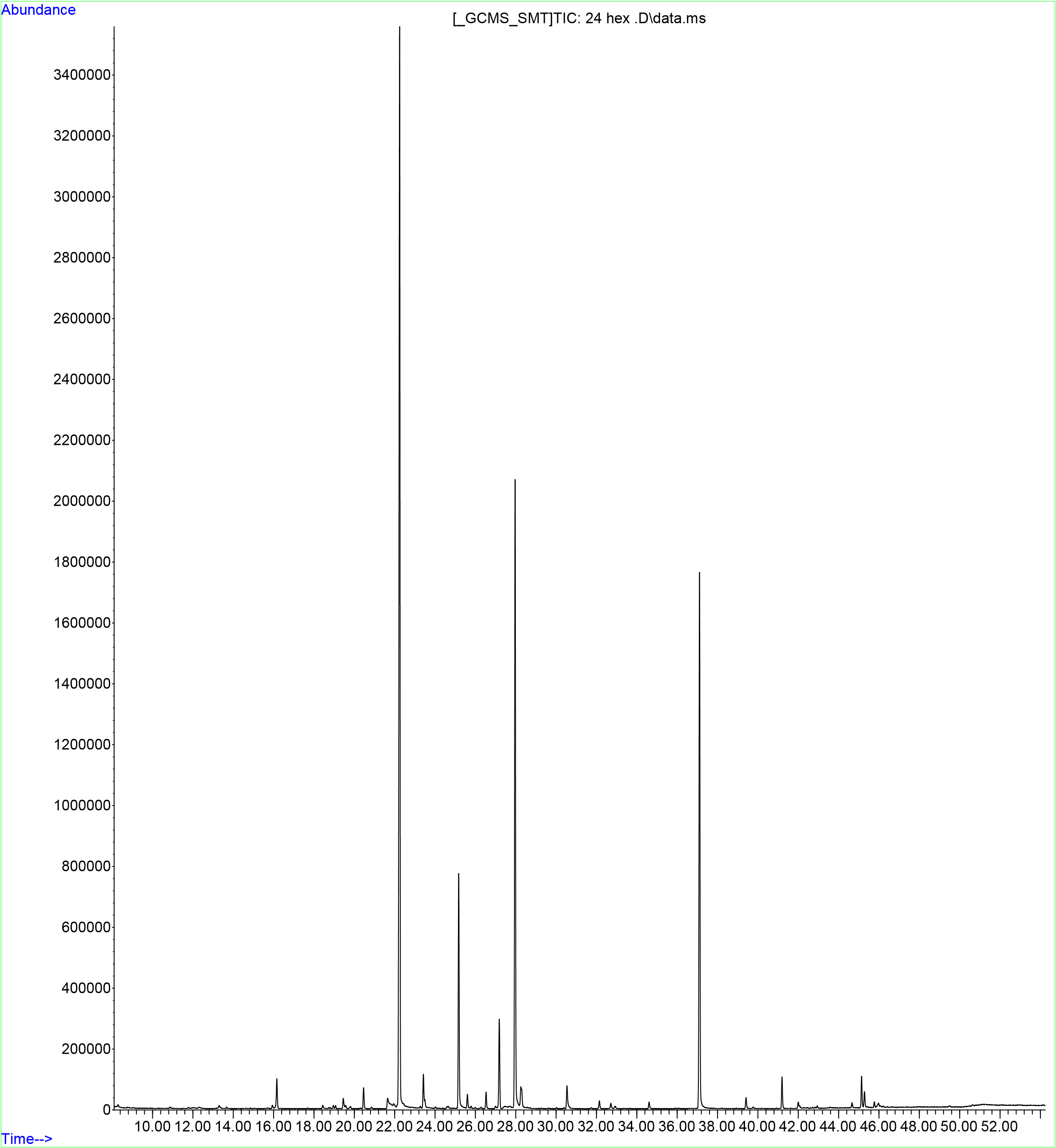


***Figure 14:*** *GCMS profile of C. zeylanicum (Sri Gemunu variety) by solvent extraction*
